# Orai3 exacerbates apoptosis of lens epithelial cells by disrupting Ca^2+^ homeostasis in diabetic cataract

**DOI:** 10.1002/ctm2.327

**Published:** 2021-03-04

**Authors:** Yong Wang, Suwen Bai, Ru Zhang, Lin Xia, Linghui Chen, Jizheng Guo, Fang Dai, Juan Du, Bing Shen

**Affiliations:** ^1^ Department of Ophthalmology The First Affiliated Hospital of Anhui Medical University Hefei China; ^2^ Department of Physiology, School of Basic Medicine Anhui Medical University Hefei China; ^3^ Department of Endocrinology The First Affiliated Hospital of Anhui Medical University Hefei China

Dear Editor,

Calcium participates in many important physiological processes.^1^ Previous reports indicate that store depletion‐operated Ca^2+^ entry (SOCE) is one of the most common and ubiquitous pathways for Ca^2+^ influx.^2^ Orai family proteins localize at the plasma membrane to form a type of SOCE channel. The depletion of Ca^2+^ stores evokes Ca^2+^ influx via SOCE channel.^3^ Three members of the Orai protein (Orai1,2,3) display high selectivity for Ca^2+^.^4^ Stromal‐interacting molecule (STIM) family proteins, sensors of Ca^2+^ depletion in the lumen of the endoplasmic reticulum (ER), rapidly translocate into ER–plasma membrane junctions to tether and activate Orai channels.^3^

Diabetes mellitus is a severe metabolic disease with a number of accompanying complications.^5^ Cataract is the major cause of blindness worldwide, and any degree of opacity in the lens is referred to as a cortical or posterior subcapsular cataract,^6^ the most common cataract observed in patients with diabetes.^7^ Here, we offer a novel mechanism for apoptosis occurring in a lens epithelial cell line from human (HLEpiC), hypothesizing that the enhanced apoptosis of lens epithelial cells in the cataracts of patients with diabetes is related to excess influx of Ca^2+^ into lens epithelial cells via Orai3 channels located in the plasma membrane.

Here, we used immunohistochemical analysis to compare the expression levels of Orai1‐3 and STIM1‐2 proteins in lens epithelium of senile cataracts patients with or without diabetes, and found that Orai3 and STIM1 expression levels were significantly increased in senile cataracts from patients with diabetes compared with those without diabetes (Figure S1, Table S1). Next, we used high glucose medium to mimic diabetic hyperglycemia in vitro. HLEpiCs were cultured with normal glucose (5.5 mM) or high glucose (25.6 mM) for 1, 3, 7, and 14 days and found that compared with the cells in normal glucose, the Ca^2+^ influx via SOCE was significantly increased in the high glucose group after either thapsigargin (TG) or ATP treatment, and the increase in SOCE was greater when the high glucose treatment lasted longer (Figure 1). Moreover, compared with those in the cells in normal glucose, the expression levels of Orai3 and STIM1 proteins were significantly enhanced in the cells in high glucose on each day (Figure S2A, B, and E). By contrast, Orai1 protein levels were decreased on each day in cells cultured in a high glucose medium compared with those in normal glucose medium (Figure S2C). The expression level of Orai2 protein in the high glucose samples was similar to that in the normal glucose samples on each day (Figure S2D).

**FIGURE 1:**
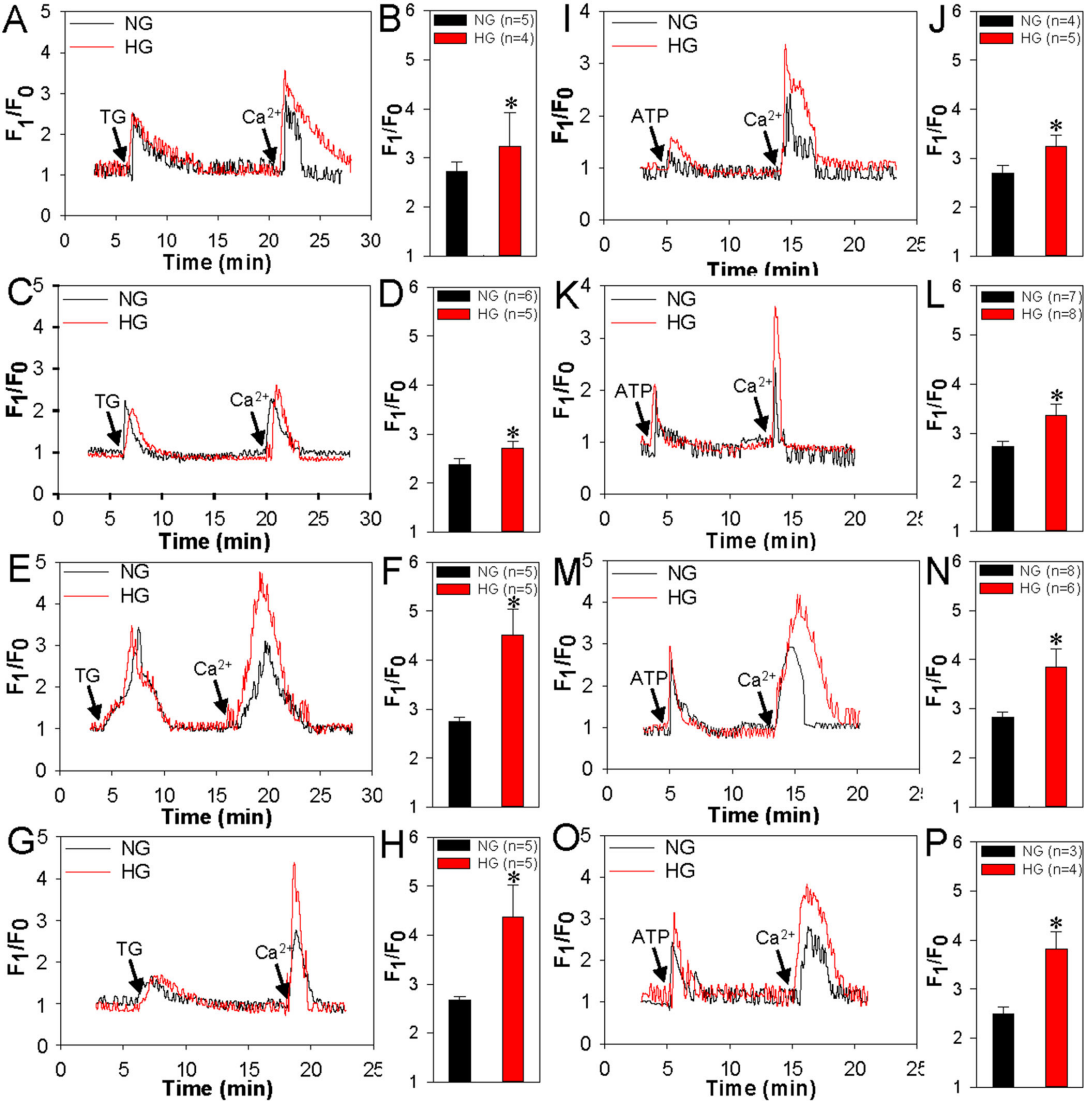
Changes of thapsigargin (TG)‐ and ATP‐induced store‐operated Ca^2+^ entry (SOCE) in HLEpiCs cultured in high glucose medium. Representative traces (A, C, E, G, I, K, M, and O) and summarized data (B, D, F, H, J, L, N, and P) showing the changes in the intracellular Ca^2+^ concentration of HLEpiCs cultured in normal glucose (NG, 5.5 mM glucose and 20 mM mannitol) or high glucose (HG, 25.6 mM glucose) media for 1 (A, B, I, and J), 3 (C, D, K, and L), 7 (E, F, M, and N), and 14 (G, H, O, and P) days. TG (2 μM, A–H) or ATP (100 μM, I‐‐P) was used to release or deplete the intracellular Ca^2+^ stores in HLEpiCs. Subsequent application of 2 mM Ca^2+^ evoked SOCE. Values are shown as the mean ± SEM. *n* = 3–8. *p < 0.05 versus the control (NG) group by two‐tailed Mann–Whitney *U* test

To explain the functional role of Orai3 and STIM1 proteins in SOCE of HLEpiCs, we used Orai3‐ and STIM1‐specific siRNAs to inhibit the respective proteins. The ATP‐ and TG‐induced SOCEs in HLEpiCs, which were transfected with Orai3 siRNA (Figure S3A–D) or STIM1 siRNA (Figure S3E–H), were markedly decreased compared with those in the control group (cells transfected with scrambled siRNA). Additional HLEpiCs were cultured in high glucose medium for 7 days and then transfected with Orai3‐ or STIM1‐specific siRNA. Compared with that in the control group, the ATP‐ and TG‐induced SOCEs were decreased in cells which were transfected with Orai3 siRNA (Figure S3I–L) or STIM1 siRNA (Figure S3M–P).

Abnormal apoptosis of lens epithelial cells is related to the development of diabetic cataract (DC).^8^ Therefore, we further assessed the changes in apoptosis of HLEpiCs in a high glucose environment and evaluated the effects of Orai3 on the apoptosis of HLEpiCs under various conditions. Our data suggested enhanced apoptosis of HLEpiCs (Figure S4A–D). The TUNEL assay showed that the ratio of apoptotic cells was enhanced in the high glucose environment compared with that in the normal glucose environment (Figure S4E–F).

To confirm the potential apoptotic effects of Orai3, we transfected HLEpiCs with Orai3 siRNA and found that Orai3 siRNA significantly increased Bcl‐2 expression, but decreased Bax expression. The slight increase in the expression level of caspase‐3 protein in response to Orai3‐specific siRNA treatment was not statistically significant (Figure S5A, C, D, and E). But in high glucose conditions, Orai3‐specific siRNA significantly increased Bcl‐2 but decreased both Bax and cleaved caspase‐3 protein expression levels (Figure S5B, F, G, and H). These results suggested that a high glucose environment significantly stimulated HLEpiCs apoptosis, and Oria3 may play a crucial role in the process.

To confirm the role of Orai3 in the pathogenicity of DC, we knocked out *Orai3* gene in SD rats. Our results indicated that aquaporin‐3 and connexin‐46 proteins as lens epithelial cell biomarkers are expressed in the primary cultured lens epithelial cells from *Orai3^–/–^* rats (Figure 2A).^9^ Ca^2+^ measurement showed that TG‐evoked SOCE was nearly abolished in the primary cultured lens epithelial cells of *Orai3^–/–^* rats compared to the cells of wild‐type rats (Figure 2B and C). We then injected streptozotocin into the rat abdomen to induce DC model. The fasting blood glucose levels were significantly higher both in streptozotocin‐injected *Orai3^–/–^* and wild‐type rats compared to control *Orai3^–/–^* and wild‐type rats (Figure 2D–G). Moreover, the lens turbidity levels were markedly higher both in diabetic *Orai3^–/–^* and wild‐type rats compared to control *Orai3^–/–^* and wild‐type rats (Figure 2H–I), but interestingly, the lens turbidity levels were significantly lower in *Orai3^–/–^* diabetic rats compared with wild‐type diabetic rats (Figure 2H–I).

**FIGURE 2:**
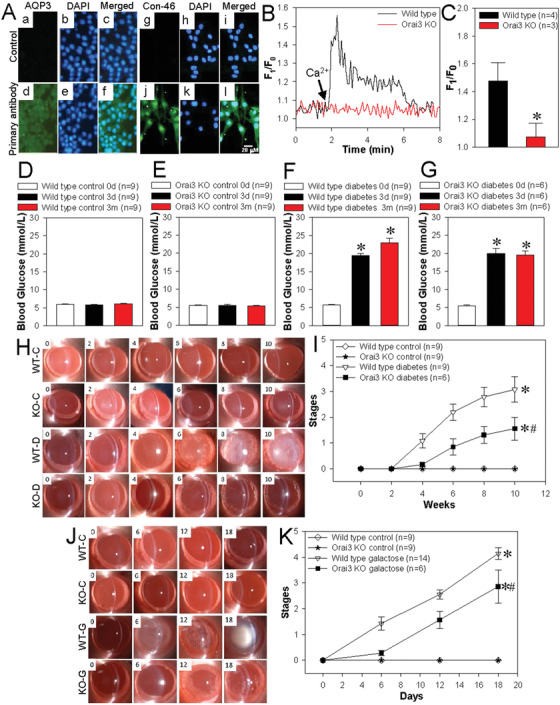
Role of Orai3 in diabetic cataract development. (A) Immunofluorescence images showing the expression of aquaporin‐3 (AQP3) or connexin‐46 (Con‐46) protein in primary cultured lens epithelial cells from *Orai3^–/–^* rat. (a‐‐c, g–i), No primary antibody control. (d–f) AQP3 expression. (j‐‐l) Con‐46 expression. The cellular nucleus was stained by DAPI. Representative traces (B) and summarized data (C) showing store‐operated Ca^2+^ entry‐induced intracellular Ca^2+^ increase of the primary cultured lens epithelial cells from wild‐type and *Orai3^–/–^* (Orai3 KO) rats. Values are shown as the mean ± SEM. *n* = 3–4. *p < 0.05 versus wild type by two‐tailed Mann‐Whitney *U* test. (D–G) The fasting blood glucose at 0 day (0 d), 3th day (3 d), 3th month (3 m) after streptozotocin (STZ) (diabetes) or solvent (control) intraperitoneal injection in the wild‐type and *Orai3^–/–^* rats. Values are shown as the mean ± SEM. *n* = 6–9. *p < 0.05. 3 d or 3 m versus 0 d by two‐tailed Mann–Whitney *U* test. Representative images (H) and summarized data (I) showing the lens turbidity stages at 0, 2, 4, 6, 8, and 10 weeks in STZ‐induced diabetic or control wild‐type and *Orai3^–/–^* rats. WT‐C: wild‐type control; KO‐C: Orai3 KO control; WT‐D: wild‐type diabetes; KO‐D: Orai3 KO diabetes. Representative images (J) and summarized data (K) showing the lens turbidity stages at 0, 6, 12, and 18 days in galactose‐ or solvent control‐fed wild‐type and *Orai3^–/–^* rats. WT‐C: wild‐type control; KO‐C: Orai3 KO control; WT‐G: wild‐type galactose; KO‐G: Orai3 KO galactose. Values are shown as the mean ± SEM. *n* = 6–14. *p < 0.05 diabetes versus control; ^#^ *p* < 0.05 Orai3 KO diabetes or galactose versus wild‐type diabetes or galactose by two‐way analysis of variance followed by Games–Howell *post hoc* tests

The major characterization of diabetes is hyperglycemia. We fed the rats with galactose to increase the blood sugar concentration to induce sugar cataract animal model as well.^10^ Our data showed that the lens turbidity levels were markedly higher both in galactose‐fed *Orai3^–/–^* and wild‐type rats compared to control *Orai3^–/–^* and wild‐type rats (Figure 2J–K), but similar to diabetic animal model, the lens turbidity levels were significantly lower in galactose‐fed *Orai3^–/–^* rats compared to galactose‐fed wild‐type rats (Figure 2J–K). Therefore, the results in animal models strongly suggest that Orai3 may be importantly involved in the development of DC.

In diabetes, a number of cellular pathologies are associated with increased extracellular glucose. Glucose can be transferred to sorbitol by aldose reductase, increasing cell osmosis and swelling in the lens. In our study, we provided evidence that a high glucose environment increased the apoptotic ratio of the lens epithelial cells, which would contribute to lens opacities. Moreover, we showed that this apoptosis in the lens epithelial cells was related to enhanced SOCE via Orai3 and further cytosolic Ca^2+^ overload. Therefore, our finding provides a new potential pathogenic and therapeutic target in DC treatment.

In summary, we demonstrated that the expression levels of two SOCE‐related proteins, Orai3 and STIM1, were significantly enhanced in lens epithelial cells derived from patients with diabetes and in high glucose‐cultured HLEpiCs. Furthermore, this enhanced SOCE contributed to abnormal cellular Ca^2+^ homeostasis/signaling and Ca^2+^ overload, which in turn induced the apoptosis of lens epithelial cells and the development of DC. This is the first evidence indicating a pathological role of Orai3 in diabetic cellular disorder and complication suggesting that SOCE may be a valuable therapeutic target in DC.

## ETHICS APPROVAL

All animal experiments were conducted in accordance with the permission of the Animal Ethics Committee of Anhui Medical University. Human specimens were collected with written informed consent from each participating patient. The procedures were performed in line with the Declaration of Helsinki and Good Clinical Practice.

## DATA AVAILABILITY STATEMENT

All the data obtained and/or analyzed associated with the current study were available from the corresponding authors upon reasonable request.

## CONFLICT OF INTEREST

The authors declare that there is no conflict of interest.

## Supporting information



Supporting Information

Supporting Information

Supporting Information
