# Supplementary material for: Orai3 exacerbates apoptosis of lens epithelial cells by disrupting Ca2+ homeostasis in diabetic cataract
Source: Clin Transl Med. 2021 Mar 4;11(3):e327. doi: 10.1002/ctm2.327 (PMC7933019; doi:10.1002/ctm2.327)
Supplement: Supplementary file 1 — Supporting Information [file CTM2-11-e327-s001.docx]

**SUPPORTING INFORMATION**

**Orai3 exacerbates apoptosis of lens epithelial cells by disrupting Ca^2+^ homeostasis in high glucose environment**

Yong Wang^1,#^, Suwen Bai^2,#^, Ru Zhang^1,#^, Lin Xia^1^, Linghui Chen^1^, Jizheng Guo^2^, Fang Dai^3^, Juan Du^2^, Bing Shen^2,*^

^1^Department of Ophthalmology, the First Affiliated Hospital of Anhui Medical University, Hefei 230022, China

^2^School of Basic Medicine, Anhui Medical University, Hefei, Anhui 230032, China

^3^Department of Endocrinology, the First Affiliated Hospital of Anhui Medical University, Hefei 230022, China

**1. Supplemental Table**

**Table S1.** Age, gender and diabetic history of patients used in immunohistochemical analysis.

| Items | Senile cataracts  (Mean ± SEM) | Senile cataracts with diabetes  (Mean ± SEM) |
| --- | --- | --- |
| Age (Year) | 72.7 ± 3.2 | 71.8 ± 3.9 |
| Height (Meter) | 168.2 ± 2.5 | 171 ± 2.8 |
| Weight (Kg) | 61.7 ± 3.0 | 64.2 ± 3.3 |
| Gender | Male (9)  Female (9) | Male (5)  Female (7) |
| Years of diabetes | — | 12.9 ± 2.1 |
| Treatment  (Intraocular lens implantation) | Left (8), Right (10) | Left (7), Right (5) |

**2. Supplemental Figures**


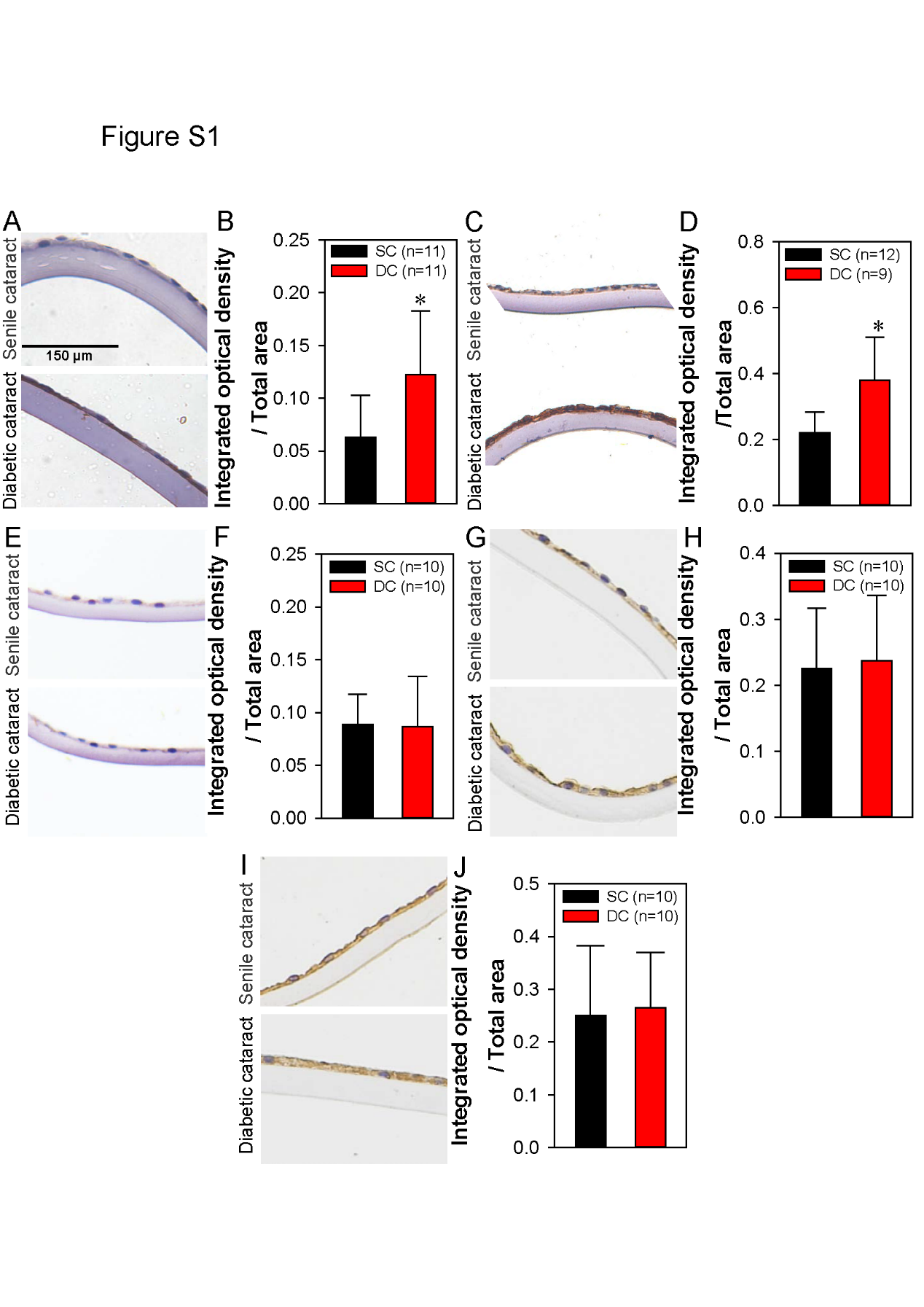


**FIGURE S1** Expression profile of Orai1-3 and STIM1-2 in human lens epithelial cells in senile cataract and senile diabetic cataract. **A**, **C**, **E**, **G** and **I**. Representative images showing Orai3 (**A**), STIM1 (**C**), Orai1 (**E**), Orai2 (**G**) and STIM2 (**I**) expression levels in human lens epithelial cells from the patients having senile cataract and senile diabetic cataract. **B**, **D**, **F**, **H** and **J**. The staining results were normalized to the optical density of the total area. Summarized data showing the ratio of integrated optical density/total area of Orai3 (**B**), STIM1 (**D**), Orai1 (**F**), Orai2 (**H**) and STIM2 (**J**) in human lens epithelial cells from patients with senile cataract (SC) and senile diabetic cataract (DC). Values are shown as the mean ± SEM. n = 9-12. **P* < 0.05. vs. the control (SC) group by two-tailed Mann-Whitney U test.


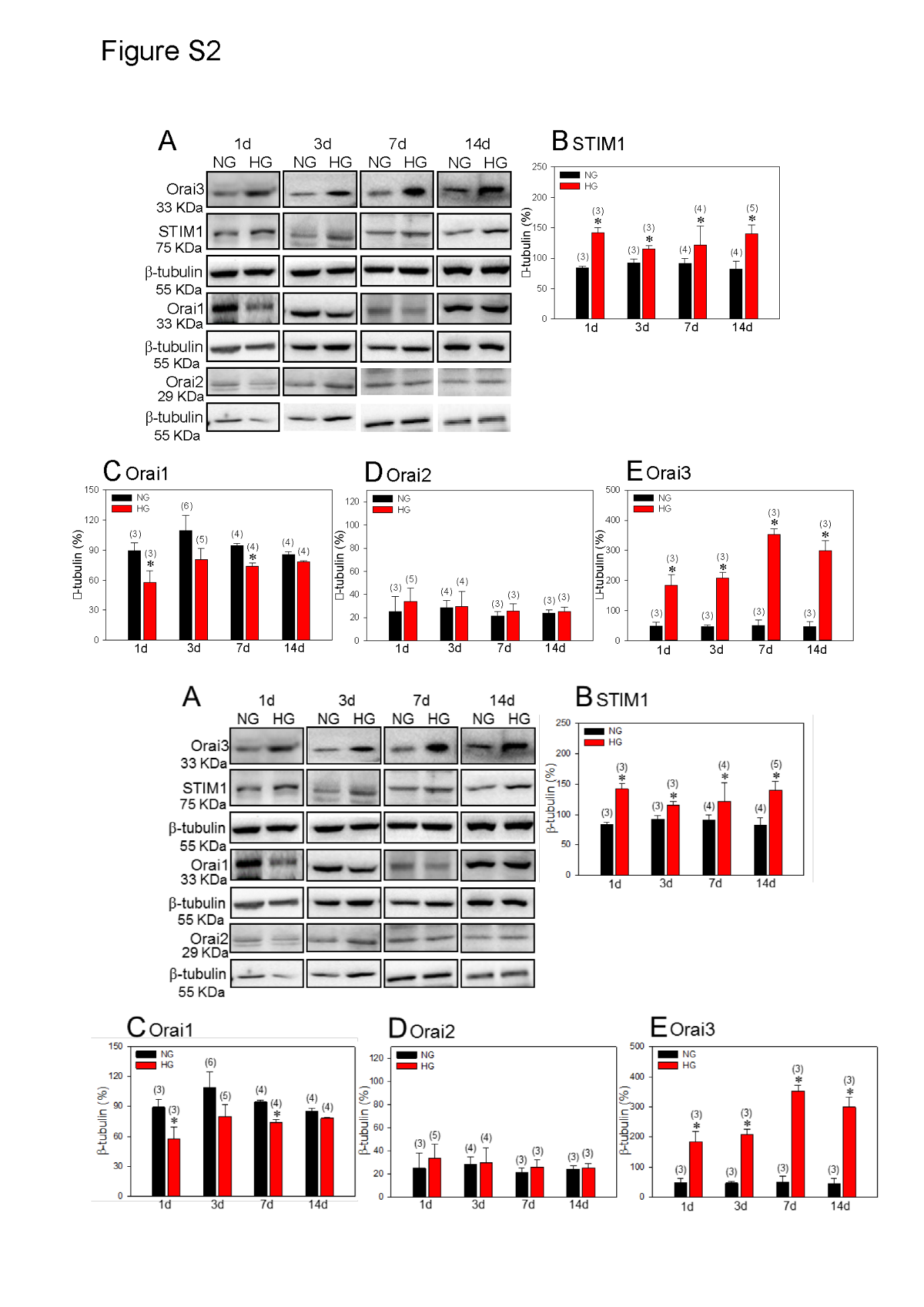


**FIGURE S2** Expression profiles of Orai and STIM1 proteins in HLEpiCs cultured in normal or high glucose media. Representative Western blotting images (**A**) and summarized data (**B**-**E**) showing the expression levels of STIM1 (**A**-**B**), Orai1 (**A** and **C**), Orai2 (**A** and **D**) and Orai3 (**A** and **E**) in HLEpiCs cultured in normal glucose (NG, 5.5 mM glucose and 20 mM mannitol) or high glucose (HG, 25.6 mM glucose) media for 1, 3, 7, and 14 days. The optical density of each protein was normalized to β-tubulin. Values are shown as the mean ± SEM. n = 3-6. **P* < 0.05. vs. the control (NG) group by two-tailed Mann-Whitney U test.


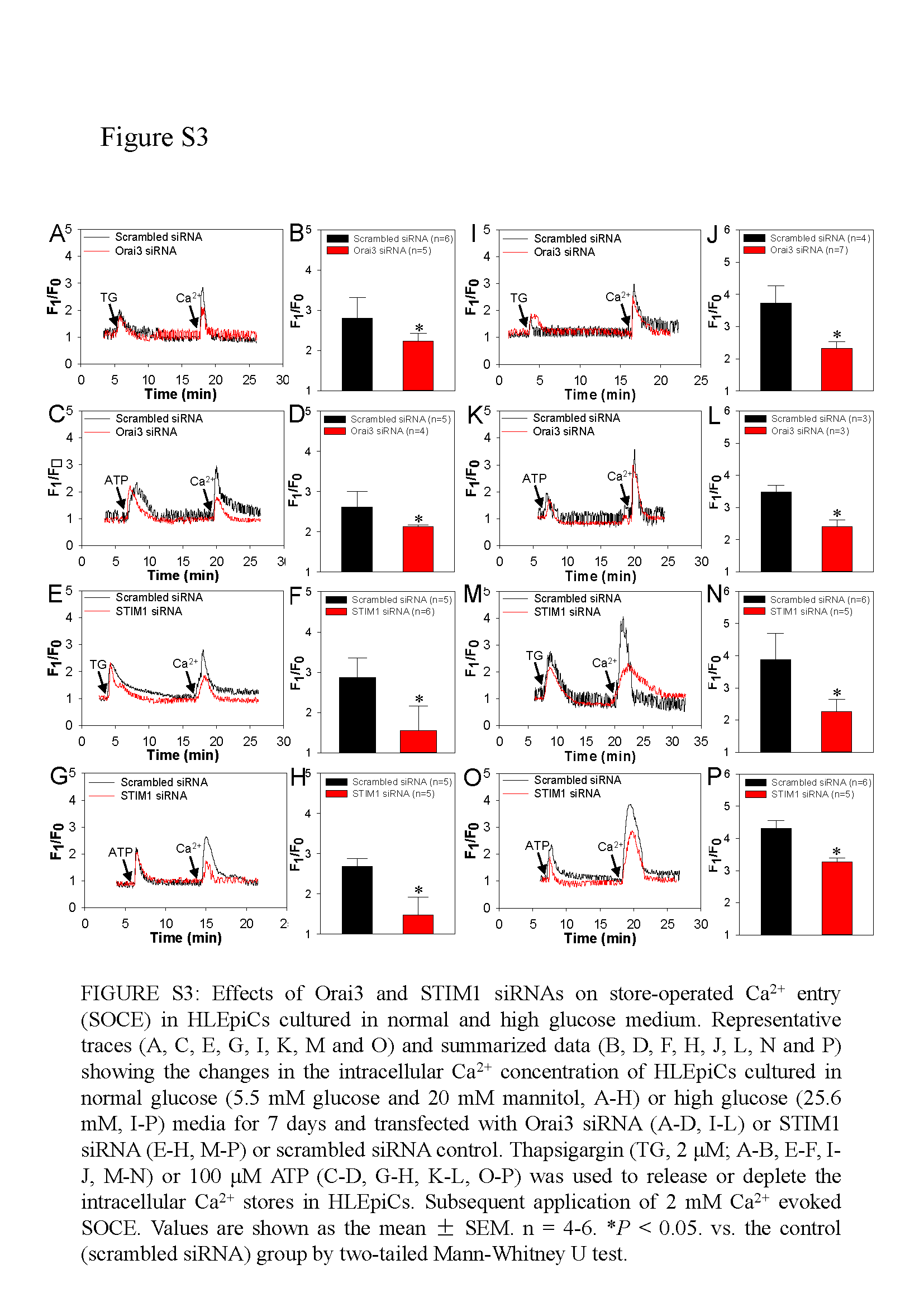


**FIGURE S3** Effects of Orai3 and STIM1 siRNAs on store-operated Ca^2+^ entry (SOCE) in HLEpiCs cultured in normal and high glucose medium. Representative traces (**A**, **C**, **E**, **G**, **I**, **K**, **M** and **O**) and summarized data (**B**, **D**, **F**, **H**, **J**, **L**, **N** and **P**) showing the changes in the intracellular Ca^2+^ concentration of HLEpiCs cultured in normal glucose (5.5 mM glucose and 20 mM mannitol, **A**-**H**) or high glucose (25.6 mM, **I**-**P**) media for 7 days and transfected with Orai3 siRNA (**A**-**D**, **I**-**L**) or STIM1 siRNA (**E**-**H**, **M**-**P**) or scrambled siRNA control. Thapsigargin (TG, 2 μM; **A**-**B**, **E**-**F**, **I**-**J**, **M**-**N**) or 100 μM ATP (**C**-**D**, **G**-**H**, **K**-**L**, **O**-**P**) was used to release or deplete the intracellular Ca^2+^ stores in HLEpiCs. Subsequent application of 2 mM Ca^2+^ evoked SOCE. Values are shown as the mean ± SEM. n = 4-6. **P* < 0.05. vs. the control (scrambled siRNA) group by two-tailed Mann-Whitney U test.


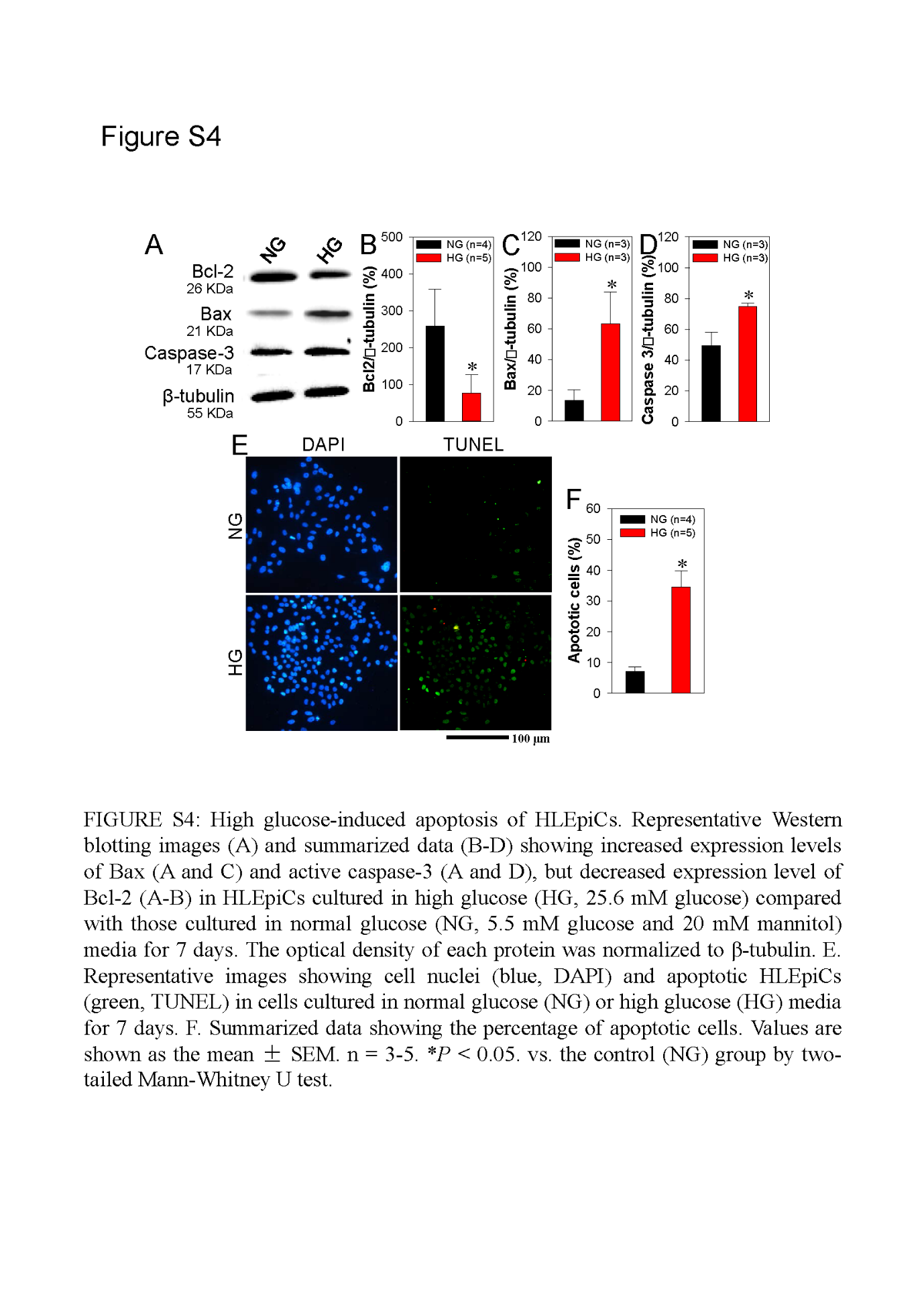


**FIGURE S4** High glucose-induced apoptosis of HLEpiCs. Representative Western blotting images (**A**) and summarized data (**B**-**D**) showing increased expression levels of Bax (**A** and **C**) and cleaved caspase-3 (**A** and **D**), but decreased expression level of Bcl-2 (**A**-**B**) in HLEpiCs cultured in high glucose (HG, 25.6 mM glucose) compared with those cultured in normal glucose (NG, 5.5 mM glucose and 20 mM mannitol) media for 7 days. The optical density of each protein was normalized to β-tubulin. E. Representative images showing cell nuclei (blue, DAPI) and apoptotic HLEpiCs (green, TUNEL) in cells cultured in normal glucose (NG) or high glucose (HG) media for 7 days. F. Summarized data showing the percentage of apoptotic cells. Values are shown as the mean ± SEM. n = 3-5. **P* < 0.05. vs. the control (NG) group by two-tailed Mann-Whitney U test.


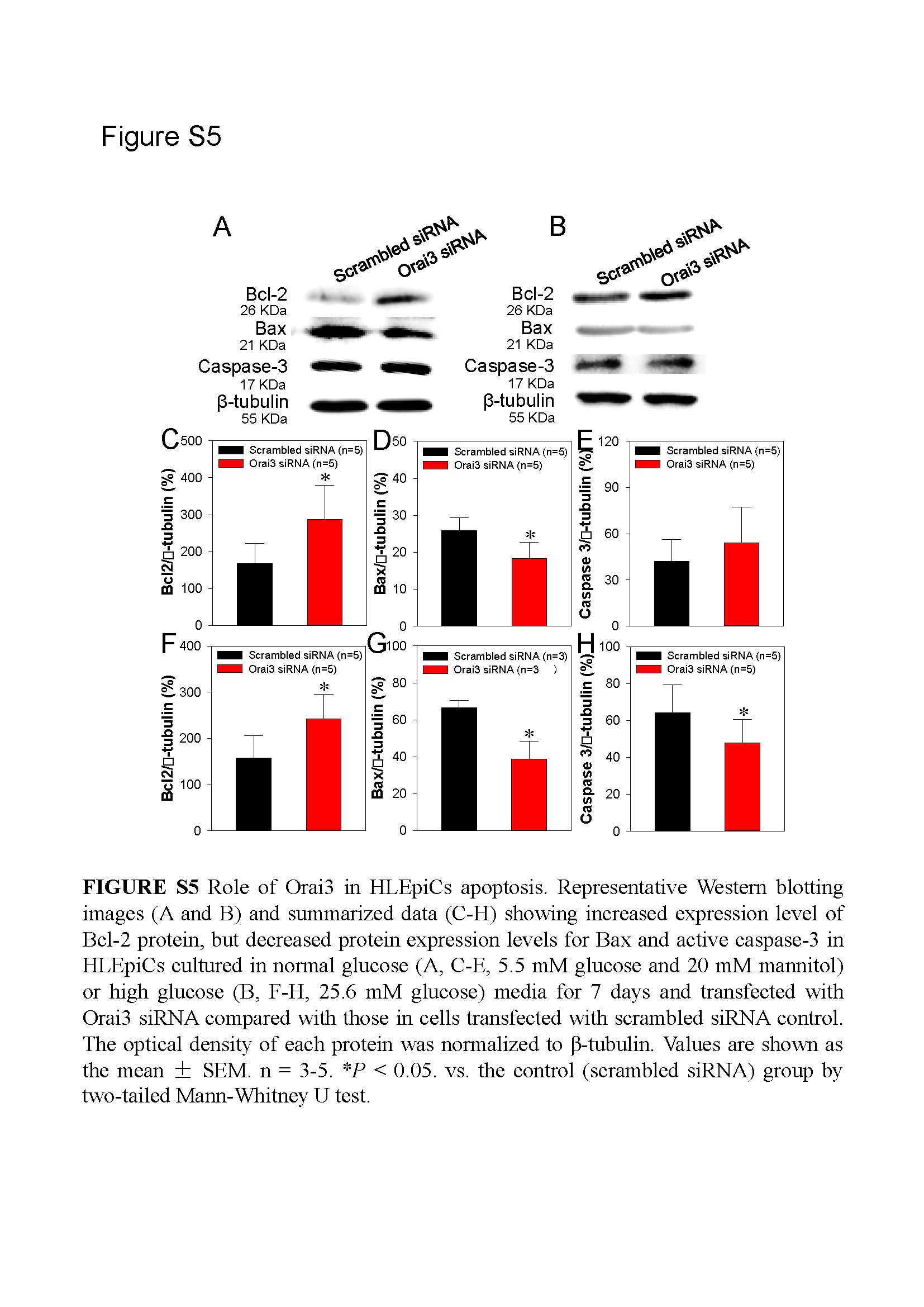


**FIGURE S5** Role of Orai3 in HLEpiCs apoptosis. Representative Western blotting images (**A** and **B**) and summarized data (**C**-**H**) showing increased expression level of Bcl-2 protein, but decreased protein expression levels for Bax and cleaved caspase-3 in HLEpiCs cultured in normal glucose (**A**, **C**-**E**, 5.5 mM glucose and 20 mM mannitol) or high glucose (**B**, **F**-**H**, 25.6 mM glucose) media for 7 days and transfected with Orai3 siRNA compared with those in cells transfected with scrambled siRNA control. The optical density of each protein was normalized to β-tubulin. Values are shown as the mean ± SEM. n = 3-5. **P* < 0.05. vs. the control (scrambled siRNA) group by two-tailed Mann-Whitney U test.


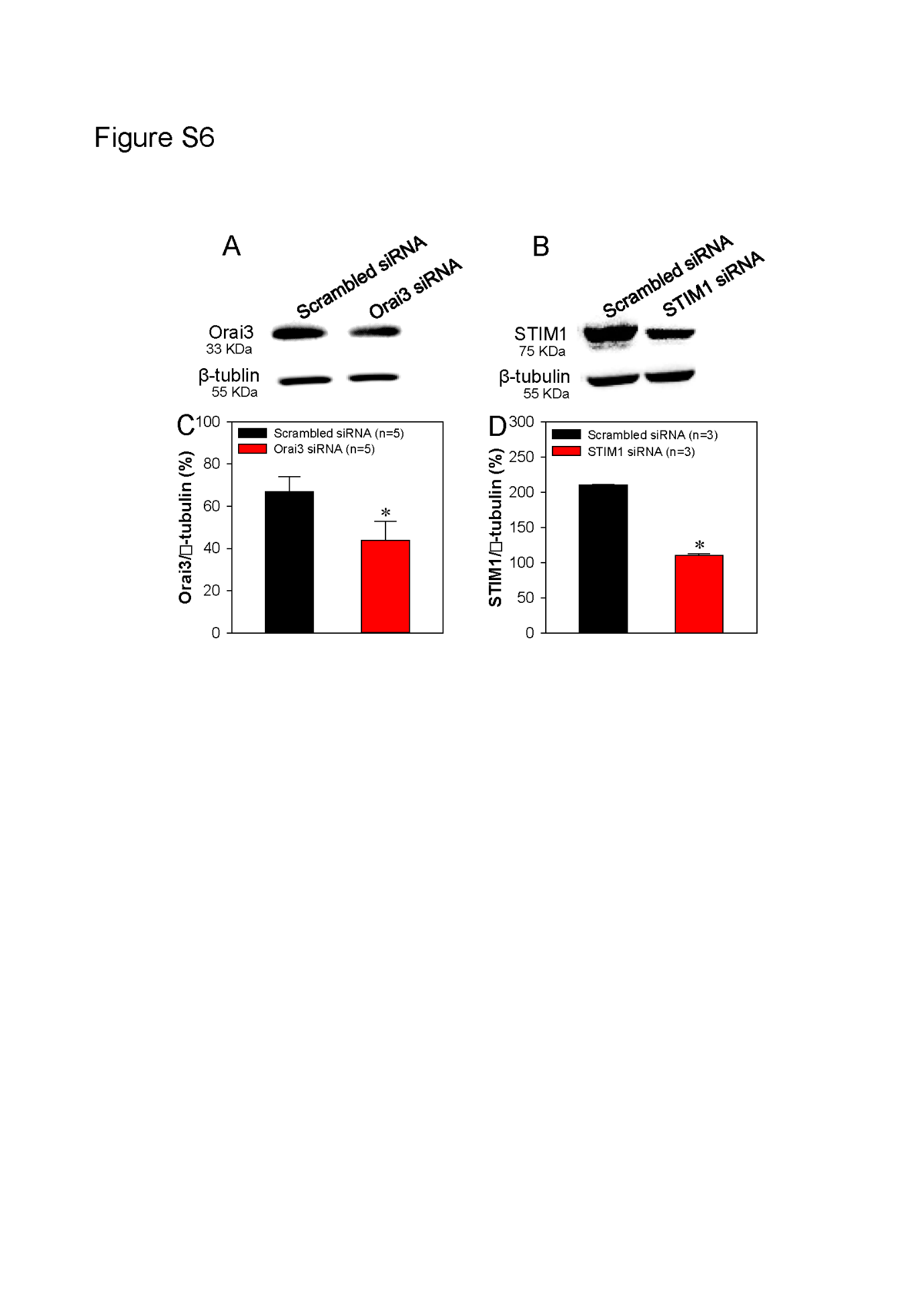


**FIGURE S6** Effects of Orai3 siRNA and STIM1 siRNA on their respective protein expression levels in a human lens epithelial cell line (HLEpiC). Representative Western blotting images (**A** and **B**) and summarized data (**C** and **D**) showing suppressed protein expression levels for Orai3 (**A** and **C**) and STIM1 (**B** and **D**) in HLEpiCs transfected with Orai3 siRNA (**A** and **C**) or STIM1 siRNA (**B** and **D**) compared with those in the scrambled siRNA control. The optical density of each protein was normalized to β-tubulin. Values are shown as the mean ± SEM. n = 3-5. **P* < 0.05. *vs*. the control (scrambled siRNA) group.

**3. Supplemental Materials and Methods**

**Materials**

ATP and thapsigargin (TG) were obtained from Calbiochem. Fluo-8 was purchased from Abcam and dissolved in DMSO. Streptozotocin (STZ), galactose were obtained from Sigma. Lipofectamine 2000 and pluronic acid (F-127) were obtained from Invitrogen. Specific siRNAs for human Orai3 (5-GGGUCAAGUUUGUGCCCAU-3)[[1](#_ENREF_1)], human STIM1 (GGUGGUGUCUAUCGUUAUU)[[2](#_ENREF_2)] and scrambled siRNA (5-ACGCGUAACGCGGGAAUUU-3) were designed and obtained from Biomics.

**Cell culture**

The HLEpiC line was purchased from the American Type Culture Collection (HB-8065, Manassas, VA, USA). HLEpiCs were maintained in Dulbecco’s modified Eagle’s medium (DMEM) supplemented with 10% FBS at 37 °C in a 5% CO_2_ incubator. The culture medium in the high glucose group contained 25.6 mM glucose, whereas the normal culture medium contained 5.5 mM glucose and 20 mM mannitol to maintain the same osmotic pressure as that in the high glucose condition. Functional studies were performed on cells cultured for 1, 3, 7, and 14 days in the two media. Specific siRNAs were transfected by Lipofectamine 2000. The transfection efficiency was determined by Western blotting assays (Figure S6).

All animal experiments were conducted in accordance with the permission of the Animal Ethics Committee of Anhui Medical University (Permission No. P2021-02-10). Wild type and *Orai3^-/-^* Sprague Dawley (SD) rats were sacrificed by inhalation of overdose CO_2_ gas. The rat eyes were isolated and the lens capsule membranes were carefully dissected out under a dissecting microscope. The isolated lens capsule membranes were quickly placed in six wells culture plate and stick to the bottom. After then, the membrane was cultured in an incubator inflated 5% CO_2_ at 37 °C for 20 min. In following 7 days culture, the lens epithelial cells gradually grew out and used in following experiments.

**Ca^2+^ measurement**

The Ca^2+^ concentration was determined as previously described[[3](#_ENREF_3)]. Cells were incubated with 10 μM Fluo-8/AM and 0.02% pluronic acid in an incubator kept in the dark for 1 h at 37 °C. The Ca^2+^ stores were depleted in cells by treating with 2 μM TG or 100 μM ATP for 10 min in Ca^2+^-free PBS (0 Ca^2+^-PBS), containing (in mM) 140 NaCl, 5 KCl, 1 MgCl_2_, 10 glucose, 0.2 EGTA, and 5 HEPES (pH 7.4). When Ca^2+^ influx was initiated, 2.0 mM extracellular Ca^2+^ was applied. Ca^2+^ fluorescence was recorded using fluorescence microscopy. Changes in the intracellular Ca^2+^ concentration ([Ca^2+^]_i_) were expressed as the ratio of fluorescence intensity before and after the extracellular Ca^2+^ application (F_1_/F_0_).

**Western blotting**

Western blotting was performed as previously described[[4](#_ENREF_4)]. The proteins were extracted with a detergent extraction buffer containing 1% Nonidet P-40, 150 mmol/L NaCl, and 20 mmol/L Tris-HCl (pH 8.0) plus protease inhibitor cocktail tablets. Total proteins (30 μg) were loaded in each well of a 10% SDS-PAGE gel. Subsequently, the proteins were transferred to a polyvinylidene difluoride membrane. After the blocking, the membrane containing the transferred proteins was incubated with respective primary specific antibodies: anti-STIM1 (1:200, rabbit polyclonal, Abcam), anti-Orai1, anti-Orai2, or anti-Orai3 (1:200, rabbit polyclonal, Santa Cruz Biotechnology, Inc.) overnight at 4 °C. The immunosignal resulting after horseradish peroxidase-conjugated secondary antibody incubation was detected using an ECL detection system. The optical intensity of the protein bands was normalized to β-tubulin, which was run on the same blots, and presented as the relative optical density.

**Immunohistochemistry**

Immunohistochemistry was performed as previously described[[5](#_ENREF_5)]. Briefly, human lens epithelial tissues from patients were obtained during clinical surgery. Specimens were collected with written informed consent from each participating patient. The procedures were performed in line with the Declaration of Helsinki and Good Clinical Practice[[6](#_ENREF_6), [7](#_ENREF_7)]. The surgical specimens were fixed with 4% paraformaldehyde and then sliced into sections 5 μm thick. The specimens were deparaffinized and rehydrated. Hydrogen peroxide (3%) incubation for 10 min was used to remove endogenous peroxidase activity. The incubation with rabbit anti-Orai3 and anti-STIM1 antibodies (1:50) was overnight at 4 °C. After secondary antibody incubation, the specimens were developed with horseradish peroxidase and then with 3,3'-diaminobenzidine tetrahydrochloride , after which the sections were counterstained with hematoxylin. For the negative control, the primary antibody was omitted.

**TUNEL assay**

The TUNEL assay was performed as previously described[[5](#_ENREF_5)]. Briefly HLEpiCs were fixed in a freshly prepared 4% paraformaldehyde solution and permeabilized by a solution (0.1% Triton X-100, 0.1% sodium citrate). Following washout and incubation in equilibration buffer, the samples were incubated in BrightGreen Labeling Mix (TdT and FITC-12-dUTP) for 1 h at 37 °C in a humidified chamber in the absence of light. The reaction was stopped by rinsing with PBS three times. The nuclei were visualized by 4',6-diamidino-2-phenylindole (DAPI) assay. Finally, the samples were examined under a fluorescence microscope. Each cell was observed for blue fluorescence (DAPI) at a wavelength of 460 nm, and the apoptotic cells were identified by green fluorescence at a wavelength of 520 nm. The percentage of apoptotic cells was calculated as follows: (apoptotic cell number/total cell number) × 100. The TdT enzyme was omitted from the labeling reaction to provide a negative control.

**Immunofluorescence staining**

The immunofluorescence staining was performed as previously described[[8](#_ENREF_8)]. Briefly, the cultured cells were seed on the coverslips, fixed, permeabilized, and blocked with bovine serum albumin. Subsequently, the cells were incubated with BSA solution (no-primary control) or anti-aquaporin-3 antibody (AQP3, 1:50, bs1253R, Bioss Company) overnight at 4°C. Following washout, the cells were incubated with secondary antibody for 1 h at room temperature. Then, the cells were washed and mounted with medium containing DAPI to stain cellular nuclear. The results were visualized using a fluorescence microscope.

**Construction of *Orai3* gene knockout rat**

SD rats were placed in a temperature-controlled room with a light-dark cycle of 12:12 h and fed with water and food freely. According to previous study[[9](#_ENREF_9)], we selected the sgRNA target on exon1 of *Orai3* gene of SD rat and designed two sgRNAs primers: sgRNA1 (5'–AGGGACGGCCCAAGTGCGGG–3'), sgRNA2 (5'–CGCGGCTACCTCGACCTTAT–3'). The sgRNA expression vector was constructed. Cas9 mRNA and sgRNA were transcribed in vitro and injected into the fertilized eggs of SD rats. Then the fertilized eggs with RNA mixture were transplanted into SD rats. The first generation was named F0 rats. The F0 rats were selected and identified by PCR (5'–CACTGGGTGGTCTGAGATCA–3', 5'–GAGCCGTGCCTACTACAACC–3') and sequencing. The positive F0 rats and wild type SD rats were bred, and the offspring was named F1 rats. Positive F1 heterozygous rats were selected by PCR and sequencing, and the offspring obtained were F2. According to PCR verification of rat genotype, *Orai3^-/-^* rat (homozygous F2) was selected.

**Establishment of diabetic rat model and classification of lens turbidity**

Diabetic rat model was established according to previous study[[10](#_ENREF_10)]. Briefly, wild type and *Orai3^-/-^* SD rats with 6 weeks old were divided into control and diabetic groups. STZ was dissolved in a citrate buffer (pH 4.2) before application. After fasting for 12 hs, the rats in diabetic or control group were intraperitoneally injected with STZ (50 mg/kg dose, one-time) or the citrate buffer as a control. After 72 hs, the tail vein blood was taken to measure blood glucose. The rats that fasting blood glucose was higher than 11. 1 mmol/L was used in diabetic groups.

After the pupils was dilated, we used a slit lamp to observe the turbidity of the lens. Cataracts are scored and recorded by an experienced ophthalmologist (the lens opacity classification standard proposed by Suryanarayana). The ophthalmologist did not know every rat identity when assessing the severity of the cataract. The development of lens turbidity is divided into five stages. Stage 0: The lens is transparent without vacuoles. Stage 1: (1) Fine vacuoles appear around the lens; (2) Vacuoles occupy 1/3 of the pro-cortex of the lens; (3) Vacuoles occupy 2/3 of the pro-cortex of the lens. Stage 2: (1) Vacuoles are liquefied or appear opalescent; (2) Very few spots and stripes of white turbidity; (3) Few white spots, strips of turbidity; (4) A lot of white spots, strips of turbidity or scattered flaky turbid. Stage 3: The lens appear scattered in the flake precipitate, the massive flake turbidity. Stage 4: Core of lens becomes turbid or most of the cortex is turbid. Stage 5: The lens is all turbid.

**Establishment of galactosemic rat model**

Galactosemic rat model was established according to previous study[[11](#_ENREF_11)]. Briefly, wild type and *Orai3^-/-^* SD rats with 6 weeks old were fed with galactosemic water or pure water. The galactose concentration was 12.5% on days 1-7 and 10% on days 8-18. Every 3-4 days, the lens turbidity was observed with a slit lamp. The degree of lens turbidity in each rat was evaluated same as the diabetic model by an experienced ophthalmologist.

**Statistical analysis**

Two-tailed Mann-Whitney U test or two-way analysis of variance followed by Games-Howell *post hoc* tests when more than two treatments were compared were performed with SigmaPlot software. Values are expressed as means ± SEM. A value of *P* < 0.05 was considered statistically significant.

**Reference**

[1] Potier M, Gonzalez JC, Motiani RK, Abdullaev IF, Bisaillon JM, Singer HA, et al. Evidence for STIM1- and Orai1-dependent store-operated calcium influx through ICRAC in vascular smooth muscle cells: role in proliferation and migration. Faseb j. 2009;23:2425-37.

[2] Jing J, He L, Sun A, Quintana A, Ding Y, Ma G, et al. Proteomic mapping of ER-PM junctions identifies STIMATE as a regulator of Ca2+ influx. Nat Cell Biol. 2015;17:1339-47.

[3] Yang Y, Zhu J, Wang X, Xue N, Du J, Meng X, et al. Contrasting Patterns of Agonist-induced Store-operated Ca2+ Entry and Vasoconstriction in Mesenteric Arteries and Aorta With Aging. Journal of cardiovascular pharmacology. 2015;65:571-8.

[4] Zhao R, Zhou M, Li J, Wang X, Su K, Hu J, et al. Increased TRPP2 expression in vascular smooth muscle cells from high-salt intake hypertensive rats: The crucial role in vascular dysfunction. Molecular nutrition & food research. 2015;59:365-72.

[5] Xue H, Lu J, Yuan R, Liu J, Liu Y, Wu K, et al. Knockdown of CLIC4 enhances ATP-induced HN4 cell apoptosis through mitochondrial and endoplasmic reticulum pathways. Cell & bioscience. 2016;6:5.

[6] World Medical Association Declaration of Helsinki: ethical principles for medical research involving human subjects. Jama. 2013;310:2191-4.

[7] Grimes DA, Hubacher D, Nanda K, Schulz KF, Moher D, Altman DG. The Good Clinical Practice guideline: a bronze standard for clinical research. Lancet (London, England). 2005;366:172-4.

[8] Shen B, Zhu J, Zhang J, Jiang F, Wang Z, Zhang Y, et al. Attenuated mesangial cell proliferation related to store-operated Ca2+ entry in aged rat: the role of STIM 1 and Orai 1. Age. 2013;35:2193-202.

[9] Xu Y, Zhao XM, Liu J, Wang YY, Xiong LL, He XY, et al. Complexin I knockout rats exhibit a complex neurobehavioral phenotype including profound ataxia and marked deficits in lifespan. Pflugers Archiv : European journal of physiology. 2019.

[10] Jiang P, Huang R, Ma N, Jiang F. The Expression of Calcium Sensing Receptor in Normal and Diabetic Rat Eyes. Medical science monitor : international medical journal of experimental and clinical research. 2018;24:706-10.

[11] Ji L, Li C, Shen N, Huan Y, Liu Q, Liu S, et al. A simple and stable galactosemic cataract model for rats. International journal of clinical and experimental medicine. 2015;8:12874-81.
