# Supplementary material for: Orai3 exacerbates apoptosis of lens epithelial cells by disrupting Ca2+ homeostasis in diabetic cataract
Source: Clin Transl Med. 2021 Mar 4;11(3):e327. doi: 10.1002/ctm2.327 (PMC7933019; doi:10.1002/ctm2.327)
Supplement: Supplementary file 2 — Supporting Information [file CTM2-11-e327-s002.pdf]

Figure S2

Orai1 1d

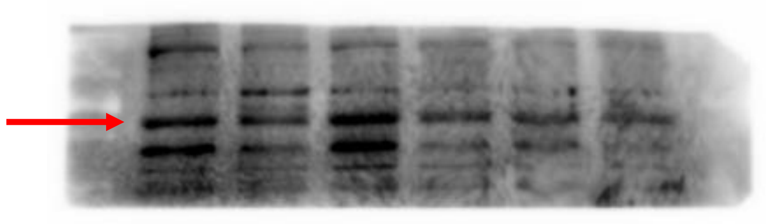

β-tubulin

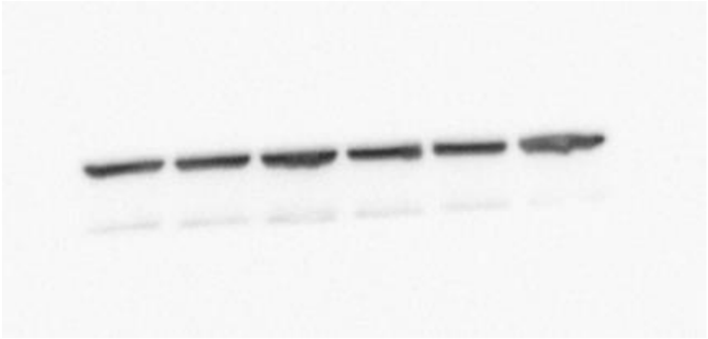

1d NG, 1d HG, 1d NG, 1d HG, 1d NG, 1d HG,

Figure S2

Orai1 3d

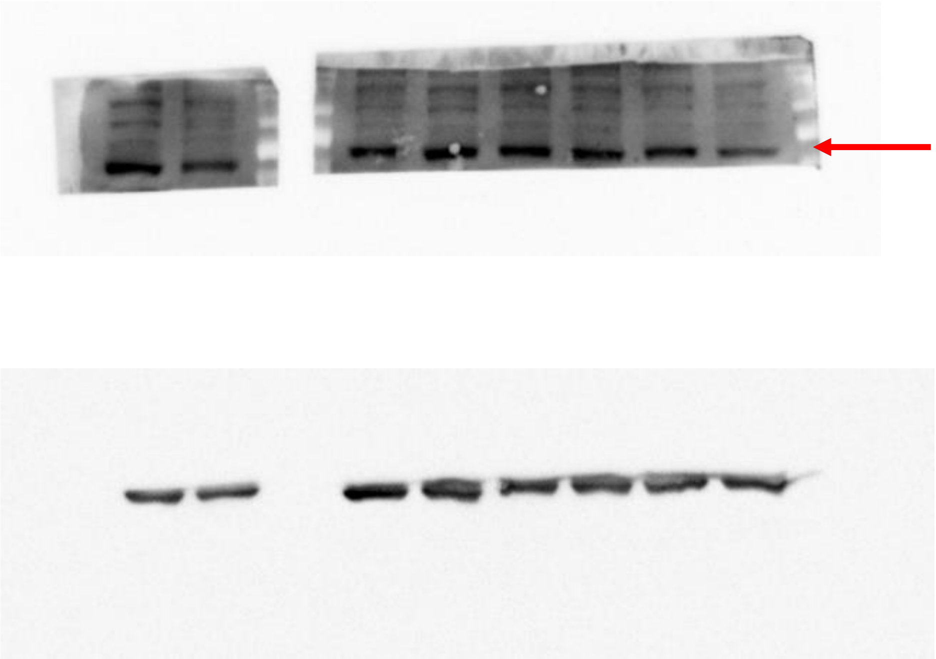

β-tubulin

1d NG, 1d HG, 1d NG, 1d HG, 1d NG, 1d HG,

Orai1 3d

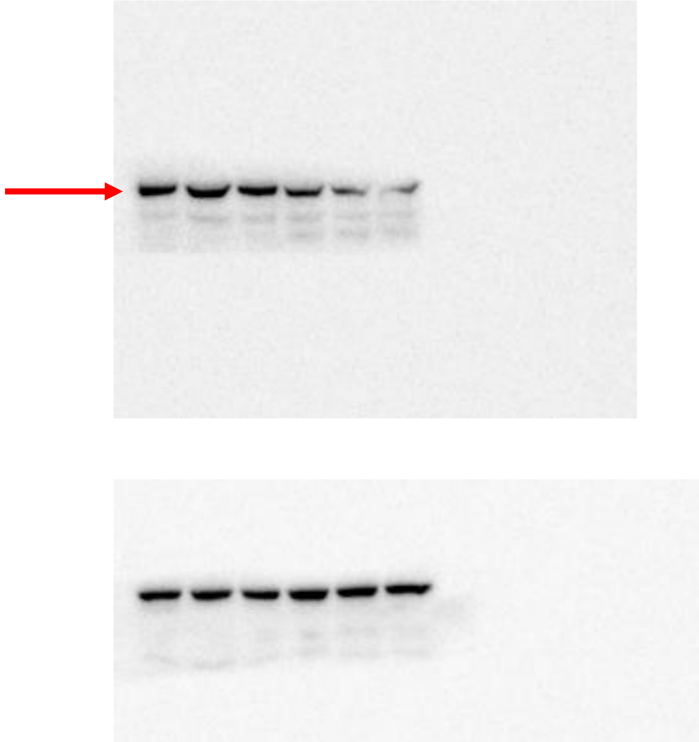

β-tubulin

1D NG, 1D NG,1D NG, 1D HG, 1D HG, 1D HG

Figure S2

Orai1 7d

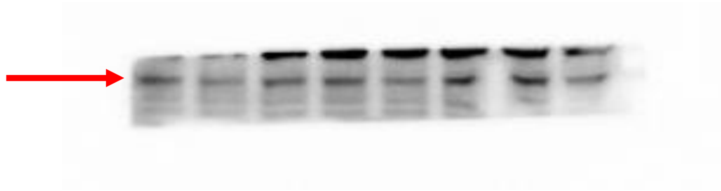

β-tubulin

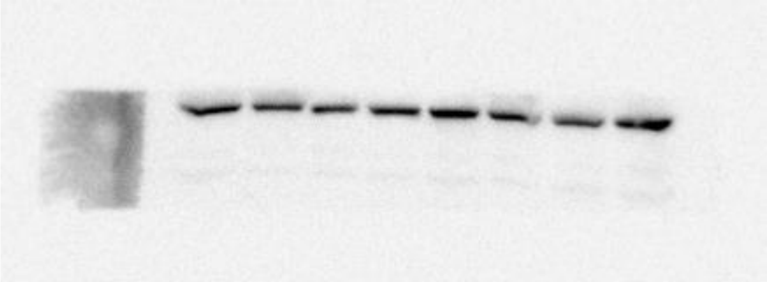

7dNG,7d NG, 7d NG,7d NG, 7d HG,7d HG, 7d HG,7d HG

Figure S2

Orai1 14d

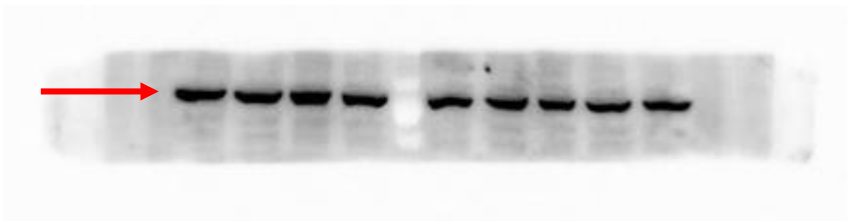

β-tubulin

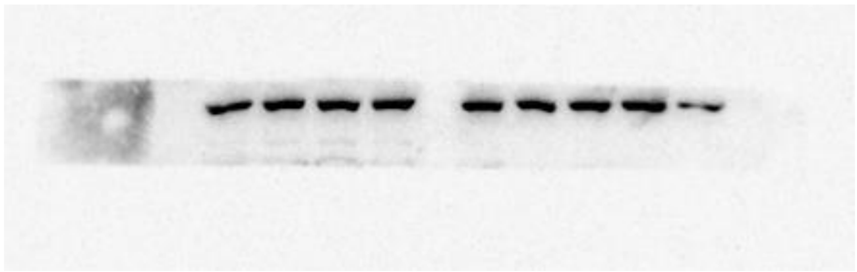

14d NG, 14d HG, 14d NG, 14d HG, marker, 14d NG, 14d HG, 14d NG, 14d HG, 14d NG,

Figure S2

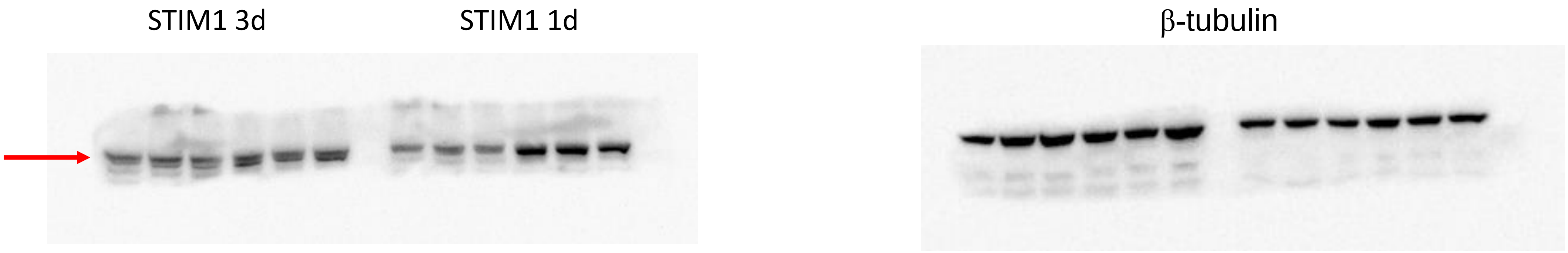

3d NG,3d NG, 3d NG, 3d HG, 3d HG, 3d HG 1d NG, 1d NG,1d NG, 1d HG, 1d HG, 1d HG

Figure S2

STIM1 7d

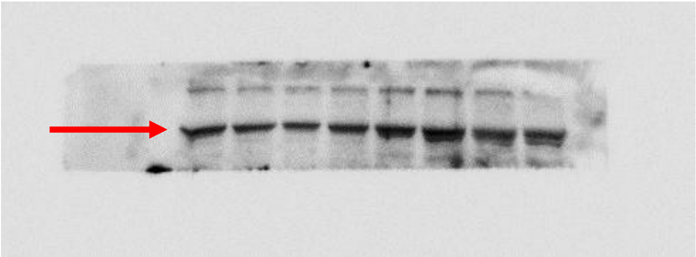

β-tubulin

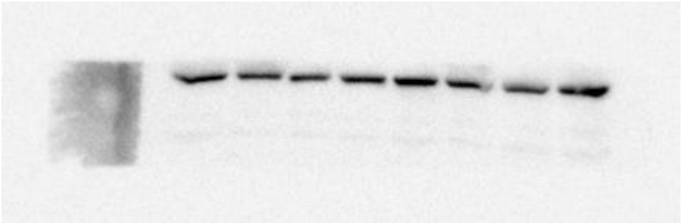

7d NG,7d NG, 7d NG,7d NG, 7d HG,7d HG, 7d HG,7d HG,

Figure S2

STIM1 14d

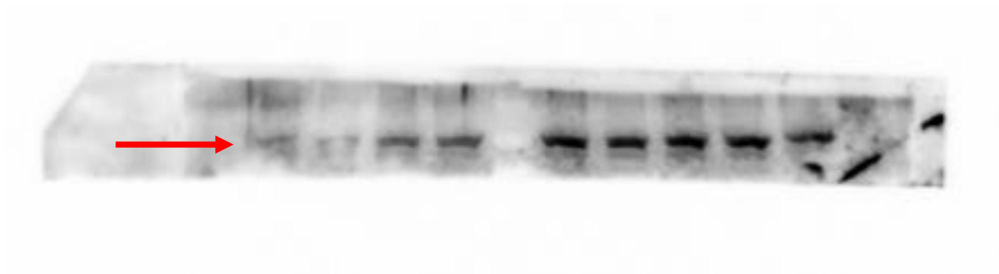

β-tubulin

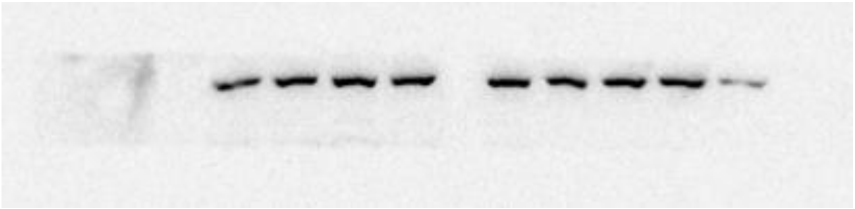

14d NG,14d NG, 14d NG,14d NG, marker, 14d HG,14d HG, 14d HG,14d HG, 14d HG

Figure S2

Orai2 1d Orai2 3d β-tubulin

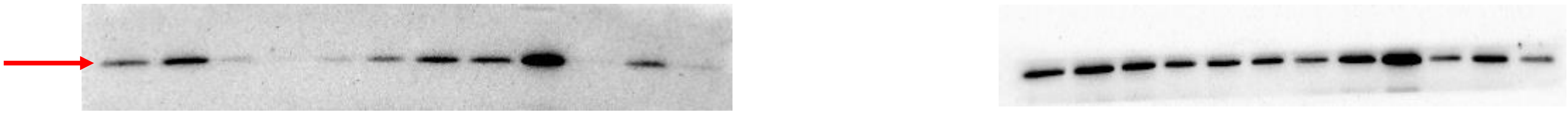

1d NG, 1d NG, 1d NG, 1d HG, 1d HG, 1d HG, 3d NG, 3d NG, 3d NG, 3d HG, 3d HG, 3d HG,

Figure S2

Orai2 1d Orai2 3d

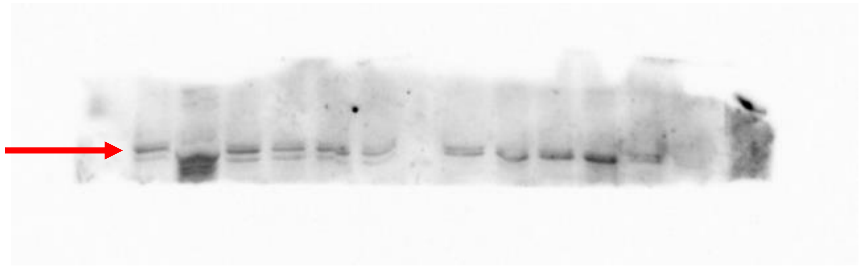

β-tubulin

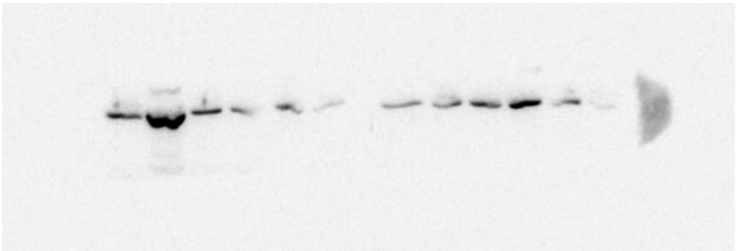

1d NG, 1d NG, 1d NG, 1d HG, 1d HG, 1d HG, 3d NG, 3d NG, 3d NG, 3d HG, 3d HG, 3d HG,

Figure S2

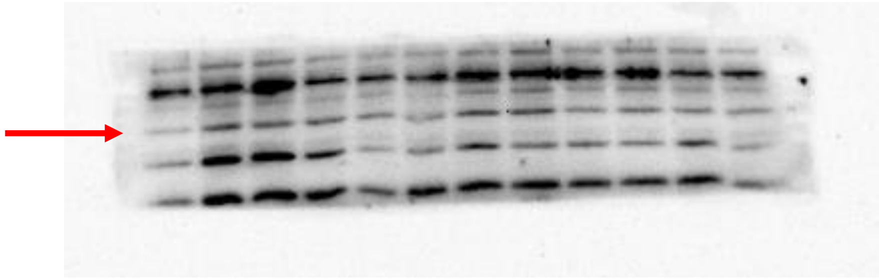

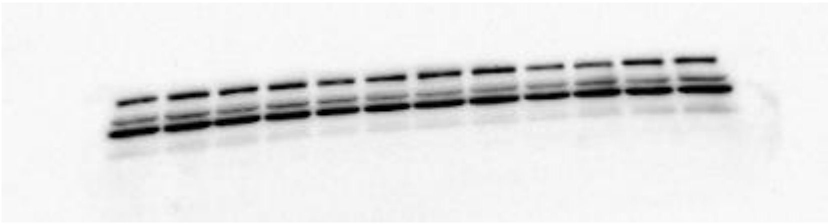

7d NG, 7d NG, 7d NG, 7d HG, 7d HG, 7d HG, 14d NG, 14d NG, 14d NG, 14d HG, 14d HG, 14d HG,
